# Managers’ support on implementation of maternal guidelines, Limpopo province, South Africa

**DOI:** 10.4102/curationis.v43i1.1949

**Published:** 2020-10-21

**Authors:** Ireen T. Ramavhoya, Maria S. Maputle, Dorah U. Ramathuba, Rachel T. Lebese, Lizzy M. Netshikweta

**Affiliations:** 1 Department of Biological Natural Science, Limpopo Nursing College, Thohoyandou, South Africa; 2 Department of Advanced Nursing Science, University of Venda, Thohoyandou, South Africa

**Keywords:** support, managers, implementation, maternal health guidelines, midwives

## Abstract

**Background:**

The report of Saving Mothers indicated a decline of maternal mortality from 12.8% to 12.5% last triennium of 2017. This shows that regardless of availability of national maternal health guidelines, midwives and managers, 25% of maternal deaths were caused by preventable and avoidable factors. As such, support provided by managers is vital in promoting the utilisation of maternal guidelines.

**Objectives:**

The objective was to determine the support offered by managers to midwives during the implementation of maternal health guidelines.

**Method:**

The study design was cross-sectional descriptive in a quantitative domain. Simple random sampling was used to select 58 operational managers and two maternal managers. Data were collected using self-administered questionnaires and analysed using Statistical Package for Social Sciences version 23. Descriptive statistics provided by Microsoft Excel in the form of charts was used to describe data. Pearson’s correlation test was used to describe relationships amongst variables.

**Results:**

The results revealed that 83.3% respondents indicated a shortage of staff to attend pregnant women. Fifty-six per cent of managers indicated that shortage of material resources contributed to substandard implementation of maternal guidelines. Supervision and monitoring of implementation of maternal guidelines was difficult as indicated by 53.3%, and 63.3% indicated lack of supervision.

**Conclusion:**

Limited support in terms of monitoring and supervision by managers was strongly indicated as having a negative effect on implementation of maternal guidelines. Capacity building was offered; however, shortage of resources led to poor implementation of maternal guidelines by midwives.

## Introduction and background

About 291 000 women die every year throughout the world because of birth-related complications (Alkema et al. 2016; WHO 2018). Most maternal deaths in developing countries are caused by preventable conditions. Maternal deaths accounted for 230/100 000 live births in 2016 compared to 16/100 000 in developed countries (UNICEF 2017). The South African Saving Mothers report of 2017 indicated a small reduction in maternal death from 12.8% in 2008–2010 to 12.5% in 2011–2013 and 2014–2016 (Department of Health 2017). Regardless of the support offered by maternal healthcare managers (MHCM) and operational managers (OMN) through supervision that promotes good practices amongst midwives, adherence to the implementation of maternal healthcare guidelines is poor. As indicated by the Saving Mothers report of 2014–2016, maternal deaths in Limpopo province stand at 165.16/1000 live births and are caused by preventable conditions and avoidable factors such as poor monitoring, incorrect management associated with failure in adhering to maternal guidelines as documented (Department of Health 2017).

Although the National Maternity Guidelines Committee at the Department of Health guided by the World Health Organization (National Department of Health [NDoH] 2016) developed the maternal health guidelines and each primary healthcare (PHC) facility has at least one or two at their disposal for midwives to utilise, the maternal mortality rate (MMR) is declining slowly by only 2.6% per year and this was far from the annual decline of 5.5% required to achieve the Sustainable Developmental Goals (Alkema et al. 2016; Say et al. 2014; WHO 2017). However, guidelines help midwives in decision-making during clinical practices and can serve as the basis for policy, planning, evaluation and quality improvement (Gagliardi et al. 2015). Irrespective of this, some of the midwives resisted changes made within the guidelines and hence managed pregnant women the way they were taught during their basic midwifery training (Ward 2014). Embo (2015) discovered that managers were supporting professional midwives by offering continuous development and, in turn, the staff was resourceful in the implementation of maternal health guidelines. Although faced with challenges such as shortage of staff, equipment and sometimes drugs, the implementation of maternal guidelines in sub-Saharan Africa and other countries between 1990 and 2013 has resulted in 50%e reduction in the levels of maternal deaths (Alkema et al. 2016; Say et al. 2014).

Despite this little progress, maternal mortality and morbidity rates are still high when compared against the departmental national target of 38/100 000. For example, 90% of women are dying from preventable maternal conditions and most of these women are from the low- and middle-income areas (Henry et al. 2018; WHO 2018). This was thought to be aggravated by inequality in the distribution of human and material resources (Alegana et al. 2015). Regardless of the challenges of resources, MHCM and OM are providing support through monthly perinatal meetings where incidences of maternal death and conditions that contributed to and/or caused mortality or morbidity are discussed and solutions are offered through capacity building (NDoH 2016).

As a form of support, the Department of Health in Limpopo province has 6776 midwives trained in essential management of obstetric emergencies (ESMOE) to ensure that midwives have the required knowledge and skills when implementing the maternal health guidelines (Department of Health 2014). Operational managers and MHCM received trainings on how to monitor, evaluate and assess whether guidelines are implemented effectively by conducting audits on maternal health records. As such, peer reviews and supervision of various PHC facilities are conducted monthly to check the quality aspects of maternal health care services by auditing maternity case records. If gaps are identified in the implementation, they draw an action plan that includes in-house in-service trainings so that they can improve the skills for implementation of maternity care guidelines by midwives. Regardless of such efforts, MMR is decreasing slowly, but in some areas it was rising (Department of Health 2017). Two districts were identified as having high maternal mortality in Limpopo province regardless of the availability of maternal healthcare guidelines, midwives and managers. As a result, the researcher asked questions on the kind of support system offered by managers that can facilitate the implementation of maternal guidelines by midwives. Hence, this study was conducted to determine the support offered by managers to midwives when implementing maternal health guidelines in Limpopo province.

## Methods and designs

### Study design

The study employed a quantitative descriptive design, in which a cross-sectional survey was conducted in order to determine the support offered by managers to midwives.

### Setting

The study was conducted from four selected municipalities of two districts in Limpopo province. The four municipalities consist of 114 PHC facilities and each is supervised by one operational manager, one assistant manager per local area, one sub-district manager per municipality and one MHCM per district. For the purpose of this study, OMNs were considered because their supervisory role is directly linked to midwives on a daily basis and MHCMs were considered as they are responsible for maternal healthcare services. The two districts had a comparatively high MMR of 242.9/100 000 live births (DoH 2017). As a result, the researcher assessed managers to determine the support they are providing to midwives during the implementation of maternal health guidelines in order to reduce MMR.

### Population and sampling

The study population comprised MHCMs and OMNs from the selected PHC facilities within the two districts. The total population was 114 and a simple random sampling was used to sample 58 OMNs and two maternal managers. The fishbowl method was used where equal numbers of small papers were folded (with either YES or NO written on them), placed in a container and mixed, and all those who chose YES were included in the study. In this study, 67 respondents chose YES; however, during data collection only 60 completed the questionnaires.

### Data collection and analysis

A self-administered questionnaire was used for data collection. The questionnaire contained closed-ended items. The questionnaire was divided into two sections: demographic characteristics and the support offered by managers to midwives during the implementation of maternal healthcare guidelines when rendering maternal healthcare services. The research team distributed questionnaires in various selected PHC facilities. Respondents completed self-administered questionnaires at their respective work place during lunch break, which took about 30 min. Data were collected from March to June 2017 because OMNs were not always available in their workplace.

#### Data analysis

Data were analysed using the Statistical Package for Social Sciences version 23 and Microsoft Excel was used to present data in the form of charts. Descriptive statistics was used to describe data and Pearson’s correlation test was used to describe the relationship amongst variables.

#### Validity and reliability

Validity and reliability were ensured before data collection of the main study. The research instrument was given to experts like the Director of Maternal Health Care who supervise and monitor the implementation of the guidelines in order to check for face and content validity. Reliability was ensured by pre-testing the instrument through five OMNs who did not form part of the main study; two questions were slightly rephrased for clarity and the Cronbach’s alpha test yielded a significant level of *p* < 0.06.

### Ethical consideration

Ethical clearance to conduct the study was obtained from the University of Venda Research Ethics Committee (SHS/16/ PBC/34/1910). The Limpopo Provincial Department of Health (Ref 4/2/2) and district executive managers granted permission to access the facilities. The voluntary nature of participation in this study and the time it took to complete a questionnaire were explained to the respondents. Respondents were assured that they can withdraw from the study at any time during data collection without being penalised. The purpose and significance of the study were explained. To ensure anonymity and confidentiality, each questionnaire had a participant code assigned by the researcher and no names or contact numbers of the respondents were written in the questionnaires. Respondents were informed that the information collected from them might be published but the individual’s identity would not be revealed. To ensure justice and fairness, respondents who met the characteristics required for this study were randomly selected. Respondents had to sign an informed consent form before completing the questionnaires.

## Presentation of results

### Demographic characteristics of respondents

As shown in Table 1, 60 managers participated in this study. A total of 58.3% (*n* = 35) acted as OMNs, 38.3% (*n* = 23) were permanently appointed and 3.4% (*n* = 2) were MHCMs. About 6.7% (*n* = 4) of respondents ranged between 30 and 39 years, 41.7% (*n* = 25) ranged between 40 and 49 years and 40% (*n* = 24) were aged over aged 50–59 years. The mean age of the respondents was 40–49 years; the researcher assumed that at this age people are more likely to be in the managerial positions in their career. With regard to gender, 80% (*n* = 57) were women and 20% (*n* = 3) were men, and the researcher observed that this was influenced by the nature of the occupation as it needed a more caring nature that is more common amongst women. With regard to years of experience as a manager, 75% (*n* = 45) of respondents had more than 5 years of experience, whereas 25% (*n* = 15) had less than 5 years of managerial experience. Almost half (*n* = 26; 43.3%) of the respondents had a diploma, 45% (*n* = 27) had a degree in nursing and 11.7% (*n* = 1.7) had other degrees, which denotes a master’s degree.

**TABLE 1:** Demographic characteristics of respondents.

Variables	No. of participants (*n* = 60)	
	*n*	*%*
**Age group (years)**		
30–39	4	6.7
40–49	25	41.7
50–59	24	40.0
> 60	7	11.7
**Gender**		
Male	3	20.0
Female	57	80.0
**Position**		
Acting operational manager	35	58.3
Operational manager	23	38.3
Maternal healthcare manager	2	3.4
**No. of years in the current position**		
1–3	6	10.0
4–5	9	15.0
> 5	45	75.0
**Highest level of education**		
Diploma	26	43.3
Degree	27	45.0
Other qualification	7	11.7

### Support offered by managers to midwives on the implementation of maternal health guidelines

Support offered by managers to midwives on the implementation of maternal health guidelines was related to training, supervision, availability of equipment, drugs, transport, guideline copies and stationery available at maternal healthcare facilities.

#### Support in relation to midwives training

The majority of participants reported being supported as a result of getting the opportunity to attend trainings (93.3%; *n* = 56); being trained when there was an update (86.7; *n* = 52) and being at par through continuous maternal guidelines updates (71.7%; *n* = 43). It was also revealed that the monthly statistics is considered for nurses to be trained (83.3%; *n* = 50) and there are monthly perinatal mortality meetings that are effective in the providing of maternal healthcare (96.7%; *n* = 58). The mean score of the support in relation to midwives training (4.32 ± 0.85) was above the normal average support score of 4.0 (see Table 3). Thus, the difference in means was significantly higher by 0.32 (95% confidence interval [CI], 0.096 to 0.5372) than a normal support for midwives with a training score of 4.0; *t*(59) = −2.874, *p* = 0.006 as indicated in Table 3.

#### Support in relation to supervision

About 63.3% (*n* = 41) participants reported that there is no supervision by district MHCMs to monitor the implementation of maternal guidelines. It was also highlighted by 53.3% (*n* = 32) of respondents that there is no supervision and monitoring conducted by OMN either (Table 2). The majority of respondents (83.3%; *n* = 50) denied the availability of two midwives in attendance of a pregnant women and about 51.7% (*n* = 31) disagreed with the fact that staff is allocated according to the needs of the facility. Most of the respondents (78.3%; *n* = 47) pointed that emergency obstetric simulation training (EOST) drills were not conducted and monitored as required, whereas (21.7%; *n* = 13) disagreed with this statement. There was a statistical difference between the support through supervision mean score (1.65 ± 1.34) and the normal score anticipated. The difference in means was significantly lower by −0.35 (95% CI, −0.6957 to −0.0043) compared to the normal average of support regarding supervision of value 2.0; *t*(59) = −2.026, *p* = 0.047 (Table 3).

**TABLE 2a:** Support offered by managers related to capacity building to human resources.

Statements	Yes		No	
	*n*	%	*n*	%
1. Do the district executive managers and operational managers release midwives without any hassles for the purpose of attending trainings?	56	93.3	4	6.7
2. Do you consider delivery statistics in order for nurses to be trained?	52	86.7	8	13.3
3. Are midwives trained if there is an updated or new guideline before implementation?	50	83.3	10	16.7
4. Is continuous education (updates) on maternal guidelines offered throughout?	43	71.7	17	28.3
5. Is the monthly perinatal mortality meeting effective in the provision of maternal healthcare?	58	96.7	2	3.3
6. Is the supervision by district maternal health managers done to monitor the implementation of maternal guidelines once in 2 months?	19	31.7	41	63.3
7. Are the supervision and monitoring of professional midwives in the implementation of maternal guidelines done daily by OMN in a health care facility?	28	46.7	32	53.3
8. Is staff allocated according to the needs of the facility?	29	48.3	31	51.7
9. Are two mid wives allocated to attend to pregnant women including at the night?	10	16.7	50	83.3
10. Are EOST drills conducted and monitored twice per month?	13	21.7	47	78.3
11. Are the criteria for transfer of women at risk and emergency known and followed by midwives?	53	88.3	7	11.7
12. Are maternal health incidences reported and investigations started within 24 h?	47	78.3	13	21.7
13. Is health education with regard to dashboard indicators given daily?	28	46.7	32	53.3

EOST, emergency obstetric simulation training; OMN, operational managers.

**TABLE 2b:** Support offered by managers related to material resources.

Statements	Yes		No	
	*n*	%	*n*	%
1. Is basic equipment such as blood pressure machine always available all the time?	17	54.0	33	55.0
2. Is emergency transport always available in case of emergency?	22	36.7	38	63.3
3. Are the ambulances’ turnaround time within norm, arrive within 60 min after a request?	16	26.7	44	73.3
4. Are emergency drugs such as magnesium sulphate always available?	57	95.0	3	5.0
5. Are uteretonics always available and used for all women who have delivered?	46	76.7	14	23.3
6. Are maternal guidelines available in each consulting room?	27	45.0	33	55.0
7. Is there a client satisfaction questionnaire/survey available in all healthcare facilities to be completed by all mothers after delivery?	16	26.7	44	73.3
8. Are clinical audits conducted on maternal records once per month?	36	60.0	24	40.0

**TABLE 3:** One-sample test on measures of support from managers.

Support in relation to	Mean ± SD	*T*	df	Sig.	Mean Dif.	95% CI of the difference	
						Lower	Upper
Midwives training	4.32 ± 0.85	2.874	59	0.006	0.32	0.0962	0.5372
Supervision	1.65 ± 1.34	−2.026	59	0.047	−0.35	−0.6957	−0.0043
Equipment	0.88 ± 0.32	−2.791	59	0.007	−0.12	−0.2003	−0.0330
Availability of drugs	1.72 ± 0.49	−4.476	59	0.000	−0.28	−0.4100	−0.1567
Availability of transport	0.63 ± 0.75	−13.959	59	0.000	−1.37	−1.5626	−1.1708
Maternal healthcare	2.58 ± 1.03	−10.655	59	0.000	−1.41667	−1.6827	−1.1506

SD, standard deviation; CI, confidence interval; T, *T*-value; df, degree of freedom; Sig., significance; Mean Dif., mean difference.

#### Support in relation to equipment

Table 2 shows that the majority of participants (88.3%; *n* = 53) agreed that the standardised eclamptic box had always been there at each and every facility. However, most of the respondents disagreed that basic equipment such as BP machines are always available at all times (55.0%; *n* = 33). There was a significant difference between the support in terms of equipment mean score (0.88 ± 0.32) and the normal average score required; thus, the mean difference was −0.12 (95% CI, −0.2003 to −0.0330); *t*(59) = −2.791, *p* = 0.007 as indicated in Table 3.

#### Support in relation to availability of drugs

The respondents agreed that emergency drugs (95.0%, *n* = 57) and uteretonics are always available and used for all women who delivered (76.7%, *n* = 46) (Table 2). There was a significant difference between the support related to availability of drugs mean score (1.72 ± 0.49) and the normal average anticipated (2.0), with a mean difference of −0.28 (95% CI, −0.4100 to −0.1567); *t*(59) = −4.476, *p* = 0.000 (Table 3).

#### Support in relation to availability of transport

As shown in Table 2, the majority of respondents reported disagreement in terms of support regarding availability of emergency transport in case of emergency (63.3%; *n* = 38) and that the ambulances turnaround times are not within the normal permissible time limit after being requested (73.3%; *n* = 44). The mean score of support in relation to availability of transport (0.63 ± 0.75) was significantly different and lower at –1.37 (95% CI, –1.5626 to –1.1708); *t*(59) = –13.959, *p* = 0.000.

#### Support in relation to maternal healthcare

Most of the participants agreed that the criteria for transfer of women at risk and emergency are known and followed (88.3%; *n* = 53) and that maternal health investigations are started within 24 h (78.3%; *n* = 47). However, most of the participants also disagreed that health education regarding dashboard indicators is given daily (53.3%; *n* = 32). There was a statistical difference between the support related to maternal healthcare mean score (2.58 ± 1.03) and the normal score anticipated. The difference in means was significantly lower by −1.42 (95% CI, −1.6827 to −1.1506) compared to the normal average of support regarding supervision with a value of 4.0; *t*(59) = −10.655, *p* = 0.000. At least 45% (*n* = 27) of respondents agreed that maternal guidelines were available in each consulting room, whereas 55% (*n* = 33) did not agree with this statement. Client satisfaction surveys were to be completed by mothers after delivery; 26.7% (*n* = 16) confirmed their availability, whereas 73.3% (*n* = 44) disagreed. At least 60% (*n* = 36) of respondents indicated conducting of clinical audits on maternal records once per month, whereas 40% (*n* = 24) disagreed with this statement.

### Correlation of demographic characteristics and the support towards implementation of maternal guidelines

Table 4 shows that support in relation to supervision had a negative significant association with years spent in a position (experience) (*r* = −0.314; *p* = 0.015), while support in relation to midwives training (*r* = 0.362; *p* = 0.004) and availability of drugs (*r* = 0.276; *p* = 0.033) was associated significantly with occupation. A moderate positive significant association was found between support in relation to availability of guidelines and supervision, and maternal healthcare (*r* = 0.490; *p* = 0.000), and a weak positive association was found between support in relation to equipment and maternal healthcare (*r* = 0.273; *p* = 0.035). A moderately strong association was found between support in relation to maternal healthcare and availability of guidelines and stationery (*r* = 0.427; *p* = 0.001), support in relation to supervision (*r* = 0.643; *p* = 0.000) and equipment (*r* = 0.258; *p* = 0.046).

**TABLE 4:** Correlations.

Variables	Age	Occupation	Years in position	Years in position	Midwives training	Supervision	Equipment	Availability of drugs	Availability of transport	Availability of maternal health care
1. Age	1	-	-	-	-	-	-	-	-	-
2. Occupation	-	−0.067	-	-	-	-	-	-	-	-
	-	0.609	-	-	-	-	-	-	-	-
3. Years in the position	0.062	−0.154	-	-	-	-	-	-	-	-
	0.638	0.241	-	-	-	-	-	-	-	-
4. Years in the position	−0.134	0.186	−0.063	-	-	-	-	-	-	-
	0.306	0.156	0.634	-	-	-	-	-	-	-
5. Midwives training	−0.044	0.362**	−0.101	0.030	-	-	-	-	-	-
	0.736	**0.004**	0.443	0.821	-	-	-	-	-	-
6. Supervision	0.159	−0.103	−0.314*	−0.162	0.232	-	-	-	-	-
	0.225	0.434	**0.015**	0.216	0.074	-	-	-	-	-
7. Equipment	−0.069	−0.104	−0.115	−0.017	−0.109	0.178	-	-	-	-
	0.603	0.427	0.381	0.899	0.405	0.174	-	-	-	-
8. Availability of drugs	0.159	0.276*	0.055	−0.122	0.218	0.105	0.002	-	-	-
	0.224	**0.033**	0.676	0.354	0.094	0.426	0.989	-	-	-
9. Availability of transport	0.042	−0.092	0.010	−0.197	−0.001	0.205	0.099	0.172	-	-
	0.753	0.483	0.939	0.131	0.995	0.115	0.452	0.190	-	-
10. Availability of guidelines and stationery	0.048	−0.017	−0.215	0.025	0.119	0.490**	0.273*	0.221	0.089	-
	0.716	0.898	0.100	0.847	0.366	**0.000**	**0.035**	0.090	0.501	-
11. Maternal healthcare	0.024	0.059	−0.193	−0.120	0.230	0.643**	0.258*	0.199	0.213	0.427**
	0.854	0.657	0.139	0.363	0.077	**0.000**	**0.046**	0.128	0.102	**0.001**

Note: Bold values indicate significant correlations.

* , correlation is significant at 0.05 (two tailed);

** , Correlation is significant at 0.01 (two tailed).

## Discussion of results

### Support offered by managers to midwives on the implementation of maternal health guidelines

To facilitate the implementation of maternal health guidelines, support offered is in the form of continuous education on new or updated guidelines and was above average as indicated by 93.3% (*n* = 56) of respondents. No recent literature was discovered indicating support offered to midwives in relation to training. However, similar to these results was a study conducted by Wallace and Kosmala-Anderson (2007), who stated that 60% of their respondents were offered continued education related to implementation of guidelines. Another study conducted on the support offered in relation to educational training indicated that the majority of midwives testified that training programmes on maternal healthcare services were well conceived and implemented, but did not indicate their results statistically (Turkmani et al. 2013). Although a study conducted by Renfrew et al. (2014) stated that guidelines implementation would be conducted by trained personnel, the significance of such information was not statistically indicated. Support offered in the form of training might have a positive impact on maternal healthcare services when midwives apply the learnt information correctly (Janmejaya 2016). As such, Jefford et al. (2018) were of the notion that the healthcare system would benefit from the application of knowledge acquired by midwives.

However, Braddick et al. (2015) argue that healthcare professionals might fail to implement the guidelines because the information was superficial and there were some discrepancies as to which guideline they were to implement. Failure to implement could lead to the provision of sub-standard care. The results revealed consensus that monthly perinatal mortality meetings were effective as an educational platform to improve the provision of maternal health. This was in line with NDoH (2015) Chapter 14 of the maternal healthcare guidelines. According to the NDoH (2016), perinatal meetings are effective forums to disseminate updates of guidelines related to either maternal or perinatal deaths. Stipulated in the guidelines are facts related to how these meetings should be conducted and relevant people or stakeholder who should attend.

The findings of the study revealed that there was reduced support in the form of monitoring and supervision of the implementation of maternal guidelines by OMNs and district MHCMs. As such, the mean score was lower than what was expected; this was evidenced by 53.3% (*n* = 32) for OMNs and 63.3% (*n* = 41) for district maternal health managers. From the observation of researchers and one of them who was an acting operational manager, it was clear that most of the time OMNs were out of their facilities attending either a workshop or a meeting that led to poor staff management. This resulted in the imparting of sub-standard care. The World Health Organization (2017) was of the opinion that this could contribute to MMR. The results were similar to findings of a study conducted on district health managers in Malawi (Bradley et al. 2013). The results indicated lack of supervision by district managers and OMNs as they were deployed to render maternal healthcare services rather than carry their daily supervision roles. The monitoring and supervision of personnel was also seen as an effective tool in the management of personnel in order to evaluate whether they were implementing guidelines correctly and effectively (Bradley et al. 2013). The findings of the study revealed that there was shortage of human resources, which had a negative effect on the implementation of maternal healthcare guidelines by midwives. As a recommendation by the Saving Mothers Report of 2011–2013, more than one midwife was required to attend to pregnant women both during the day and night (DoH 2014). This was not the case in most of the PHC facilities. Most of the time, only one midwife was available to render maternal healthcare services, especially during the night and even during the day, because of shortage of staff. This contributed to limited implementation of the maternal guidelines because of increased workload. According to Ward (2014) and Kyei-Nimakoh, Carolan-Olah and McCann (2016), shortage of staff led to an increased workload that contributed to increased MMR; hence, midwives failed to reach the Sustainable Developmental Goal numbers 3.1 and 3.2.

As part of support through supervision of midwives, ESMOE trainings were introduced as a strategy to reduce MMR (DoH 2017). The findings revealed that training on EOST drills were not conducted and monitored as required, as confirmed by 78.3% (*n* = 47) of respondents. Managers were to support midwives by monitoring the implementation of EOST drills; however, this was not done as expected because of shortage of material resources. If drills were conducted as recommended, midwives would implement the maternal health guidelines competently and with confidence, resulting in the management of preventable causes of maternal morbidity and mortality conditions, such as post-partum haemorrhage, and pre-eclampsia.

There was availability of a standardised eclamptic box in all the PHC facilities; hence, midwives were able to easily manage pregnant women with pre-eclampsia. Regardless of the availability of an eclamptic box, there was shortage of other material resources as the majority respondents (55%; *n* = 33) indicated that basic equipment like a blood pressure apparatus were always not available. The blood pressure apparatus is very important to diagnose pregnancy-induced hypertension. For midwives to provide quality care to reduce maternal morbidity and mortality rate, managers need to ensure the availability and accessibility of good quality and good standard equipment. The Department of Health (2014) supports the fact that the duty of an OMN is to ensure the availability of basic equipment, such as a blood pressure apparatus; however, they sometimes send in the requisitions for equipment without success. Shortage of equipment such as the blood pressure apparatus results in unnecessary transfers of women to other hospitals, resulting in patient overload or late detection of complicated maternal health conditions. Various authors have stated that shortage of equipment made it difficult to implement maternal guidelines (Khan, Timmings & Voger 2014); Darega et al. 2016).

The results revealed that PHC facilities were having sufficient drugs such as magnesium sulphate, as indicated by values of 95.0% (*n* = 57) and 76.7% (*n* = 46) for uteretonics. This would achieve the implementation of maternal healthcare guidelines successfully. Mangundu (2017) reported different results where 55.81% of respondents reported poor drug supply that led to patients buying drugs from the pharmacy. Again the results of this current study were in contrast to the findings of Mkoka et al. (2014), where the managers indicated that suppliers of drugs were unreliable because they would place the order but would not receive the required amount. Respondents were experiencing non-availability of uteretonic drugs. Uteretonic drugs such as ergometrine may be required in the management of post-partum haemorrhage, and misoprostol that could be used if Syntocinon was not available; these drugs were only found at the hospital.

Emergency transport at PHC was a challenge, and respondents cited the lack of emergency transport. Support in relation to availability of transport was relatively low, as indicated by a value of 63.3% (*n* = 38). These results were similar to a study conducted in a healthcare facility in Zimbabwe, where 78.3% (*n* = 47) of respondents indicated poor access of ambulance services that resulted in delayed initiation of treatment by the hospital (Mangundu 2017). The norm for the turnaround time of ambulances is 60 min (counting from the time it was requested). In this study, the respondents experienced non-compliance. During peer review evaluations of PHC facilities, ambulance turnaround time was monitored for the OMNs to draw an action plan when the ambulance services were not arriving within the stipulated time. However, sometimes there were barriers in accessing the ambulances, such as shortage, inaccessible roads and distance (Khan et al. 2014; Murima 2016). Non-compliance of ambulance turnaround times was also reported by a study where the mean time for ambulance was 4.42 ± 67 min (Goh et al. 2015).

It was noted in the results that 88.3% (*n* = 53) of respondents knew the criteria for transfer if at risk during pregnancy. Midwives were aware of communication channels and types of pregnant women who needed referral to the next level and at a specific gestational age. Urgent transfer of obstetric emergencies as well as high-risk women led to delivery of pregnant women at the hospital with a positive outcome (Goh et al. 2015; Singh et al. 2016). Untoward and successful incidences were seen as learning opportunities to respondents as investigations would be followed and contributory causes would be identified and corrective measures would be taken in the form of in-service training. However, education of pregnant women regarding dashboard indicators was not taken into account by most midwives; this needed to be strengthened, because if information was disseminated, it would prevent late booking for antenatal care. Various researchers conducted randomised control studies on the effects of health education related to self-care by pregnant women. At first, the results revealed a lack of knowledge regarding the subject matters. An assessment was carried out again after health education, which yielded effective results (Suto et al. 2017; Uebelacker et al. 2016).

The ideal clinic realisation indicated that maternity care guidelines were to be provided by MHCM and made available in every room; this should be used in case of an emergency (NDoH 2015). It was noted that guidelines were only available in the labour room and/or in one consulting room. According to Khan et al. (2014), easy dissemination of guidelines was noted in the case of midwives and they discovered them when they were to be reviewed, whereas some midwives were never aware of the existence of such guidelines. In this study, only 45% (*n* = 27) of respondents had maternal guidelines in each consulting room. However, Braddick et al. (2015) were of the opinion that other staff members at the facility were not aware of the availability of such guidelines. It was further revealed by Ramli and Purwita (2018) that even when the workshops on updated guidelines were organised, they were attended by few practitioners, with limited communication and dissemination efforts to reach all healthcare practitioners. This situation was similar to the results of the current study. Furthermore, other healthcare practitioners had negative attitudes towards updated guidelines because they perceived that updated information was disseminated by different sources differently, which caused confusion on which information was to be implemented (Ramli & Purwita 2018).

The results further revealed that surveys questionnaires related to clients satisfaction were not available at all PHC facilities. Only 26.7% (*n* = 16) of respondents completed client satisfaction survey questionnaires which would assist managers to evaluate the services rendered by midwives. Kyei-Nimakoh et al. (2016) argued that client satisfaction surveys could improve maternal healthcare services and patient care would be of a higher standard. The findings of this study revealed that clinical audits were conducted on maternal records monthly though some OMNs failed to do so as most of the time they were outside the facility attending meetings. This made it impossible for them to monitor the implementation of maternal guidelines through audits. Furthermore, there was an increased chance that the implementation of maternal health guidelines will be done correctly. According to Dixon, Karagiannidou and Knapp (2018), performance of the clinical audit was viewed as a measure employed to identify the quality of patients care in comparison to approved standards of care. Esposito and Dal Canton (2014) argued that clinical audits conducted on patient medical records were a source of information that revealed incomplete information. Clinical audits and client satisfaction surveys, if conducted, would help in identifying gaps and bring change in staff attitudes towards implementation of maternal guidelines for all pregnant women.

### Correlation of demographic characteristics and the support towards the implementation of maternal guidelines

As shown in Table 4, although support through provision of training and making sufficient drugs available was offered exceptionally, there was no correlation of support offered through supervision and years of experience in a position as a manager. Regardless of years of service, supervision rate was low. This might affect the implementation of maternal healthcare guidelines negatively as there was little supervision and monitoring of the services rendered. Even though maternal healthcare guidelines were not available in all consulting rooms, with the shortage of equipment, the support in relation to maternal healthcare was moderately offered by managers.

## Recommendations

### Human resources

Operational managers should continue sending midwives for various trainings. Both MHCMs and OMNs should formulate orientation and in-service programmes on the implementation of maternal health guidelines for all staff at the facility. In addition, all midwives at all health facilities should be sufficiently trained on maternal health guidelines. Updates and continuous training on maternal health guidelines must be conducted. Maternal healthcare managers should disseminate maternal guidelines amongst all staff members who provide maternal healthcare and promote teamwork and sharing of responsibilities of midwives within the facility. The MHCMs should request for equipment that will facilitate EOST drills and OMNs should monitor and put the reporting devices in place for drills. Maternal healthcare managers should review their visiting schedules in order to support nurses at the PHC.

Deputy OMNs must be appointed to continue supervision and monitoring in the absence of OMNs. Most of the facilities are supervised by acting OMNs; therefore, recruitment and retention strategies, focusing on rural areas in particular, should be strengthened to ensure adequate human resources for the implementation of maternal guidelines.

### Material resources

Operational managers as well as midwives must be involved in the requisition of equipment. District executive managers should purchase durable equipment regardless of price so that they can last longer. The requirements for the ideal clinic and benchmarking by OMNs on comparing with other districts to see what works before purchasing equipment should be fulfilled. Client satisfaction survey questionnaires need to be re-designed and completed during the discharge of a mother. There is a need to purchase adequate ambulances and they should be stationed nearer to each facility by the Provincial Department of Health. Roads, especially in rural areas, need to be maintained, if possible tarred, for easy access to PHC facilities. To ensure availability of drugs, standardised reporting systems need to be put in place. Maternal guidelines and relevant stationery must be available in each cubicle. Maternal healthcare managers should ensure the availability of survey forms in all PHC facilities.

## Conclusion

Support in relation to training of midwives by managers was significantly noted. Regardless of years of experience in a position as an OMN or MHCM, support in relation to monitoring and supervision of midwives and EOST drills was found to be relatively low. The support in relation to availability of drugs and an eclamptic box in each PHC facility was noted; however, the shortage of midwives and equipment was a challenge, where only one midwife was allocated especially during the night to render maternal healthcare services. The mean score in terms of support in relation to the availability of transport via ambulances was relatively low. Health education in relation to dashboard indicators, client satisfaction survey and clinical audits was partially disseminated. There is a need for managers to support midwives in order to improve maternal healthcare services. This would contribute towards the reduction of MMR by 5.5% by 2030 as required by the Sustainable Developmental Goals.
